# Treasure of the Past: IV: THE STANDARD-CELL COMPARATOR, A SPECIALIZED POTENTIOMETER

**DOI:** 10.6028/jres.105.059

**Published:** 2000-10-01

**Authors:** H. B. Brooks

## Abstract

The standard cell plays a very important role in the maintenance of the electrical units and in correlating the units of the various national laboratories. Modern standard cells have attained such a high degree of reproducibility and permanence as to warrant the use of apparatus of the utmost precision and reliability in their intercomparison. The paper describes a new potentiometer developed especially for this purpose. Although it actually measures the small difference between the known emf of a reference cell and that of the cell under test, it contains a simple mechanical computing feature which automatically adds this small difference algebraically to the emf of the reference cell and thereby indicates directly the value of the emf under measurement. The design of the instrument is such that no readjustment of its coils will be required when the impending changes in the ohm and the volt are accomplished. The new instrument has been given the distinctive name, “standard-cell comparator”.

## I. INTRODUCTION

In the maintenance of the electrical units by the national standardizing laboratories and the International Bureau of Weights and Measures very important roles are played by material electrical standards of two kinds, namely, the resistance standard and the standard cell. Not the least important of their functions is their use for correlating the basic electrical units of various countries. Many cells and coils have been transported thousands of miles in order to determine the differences existing among these units and to give a definite basis for action when readjustment should become necessary. The question of suitable instruments and technique for the accurate intercomparison of these standards is therefore a matter of great importance. Bridges of various kinds are used for comparing resistance standards with extreme accuracy, and potentiometers constitute the accepted means for the comparison of standard cells. Potentiometers of the usual forms, as made for ordinary electrical measurements, have commonly been employed for this purpose, and when of suitable design and good construction and properly maintained, give results which have met the requirements very well.

In recent years, however, the movement for the revision of the values of the electrical units has accentuated the demand for high precision and reliability in the intercomparison of primary standards. For example, it is desirable that the absolute measurements of current now under way in several national laboratories shall give results agreeing to 1 part in 100,000, when reduced to a common basis. To do this, it is desirable that each laboratory should know the values of its resistance standards and standard cells, in terms of its international ohm and volt, to 1 part in 1,000,000. This in turn means that it would be desirable to hold down fortuitous errors in the comparison of its working coils and cells with those representing its units to one or two units in the next decimal place; that is, to 1 or 2 parts in 10,000,000. The extension of the accuracy of a class of measurements by an additional decimal place requires recourse to refinements and precautions previously regarded as uncalled for.

This paper describes a special potentiometer which is used in the standard-cell laboratory of the Bureau of Standards. In its design and construction all available precautions and refinements have been utilized in order that it shall meet the exacting requirements of the new era in the history of the electrical units.

## II. METHODS USED IN COMPARING STANDARD CELLS

### 1. SUBSTITUTION METHOD

In the usual substitution method the two electromotive forces to be compared are opposed successively to a controlled potential difference. In the ordinary forms of potentiometer the control is accomplished by varying a resistance *r*, through which flows a nominally constant current *i.* Since the values of this resistance, *r*_1_ and *r*_2_, for the two cells differ only slightly, this method would be capable of very great accuracy if a sufficiently constant current were available. It is at this point that the method fails to meet the present exacting demand for accuracy. The current from a storage cell tends to decrease slowly as the discharge proceeds, and is affected by changes of the temperature of the cell. The latter effect may be reduced or avoided by thermally insulating the cells or by keeping them in a thermostat. The temperature coefficient of emf of a lead storage cell being approximately 0.01 percent per ° C, it is necessary to keep the temperature of the cell constant within 0.1° C. to keep the current constant to 1 part in 100,000, so far as temperature effects are concerned. This degree of constancy may be maintained, under favorable conditions, by frequent checking and adjusting of the current, but is inadequate for the precise comparison of standard cells, for which one is obliged to use the opposition method.

### 2. OPPOSITION METHOD

In the opposition method the two cells to be compared are connected in series with their emf’s opposing each other, and the two free terminals are joined to a potentiometer with which the small difference between the two emf’s is measured. A relatively low accuracy of measurement of this difference gives a highly accurate value for one of the cells in terms of the other. This method has been known, used, and appreciated for years, but has some minor disadvantages which have caused other methods to be preferred when the highest accuracy is not required. These disadvantages are, first, that the operator must determine which of the two cells has the higher emf, and second, that an addition or a subtraction is necessary to get the value of the emf of the unknown cell. Both of these disadvantages can be avoided in a variant of the opposition method, suggested some years ago by Wenner, in which a slide wire with two sliders was to be used. This method was not developed in detail and has not been published.

### 3. REQUIREMENTS FOR A SATISFACTORY METHOD

The following requirements were set up by the writer as necessary or desirable features in a potentiometer for highly accurate comparisons of standard cells, and the design as actually worked out includes all of these features:

- It should function on the opposition principle.
- There should be no sliding contacts in that part of the potentiometer circuit in which is set up the difference of potential which balances the difference in emf of the two standard cells under comparison.
- The potentiometer proper should be free from parasitic thermal emf to better than 0.1 *μ*v, and should contain a device for the ready detection and compensation of such unwanted emf in the galvanometer and its connections to the potentiometer.
- The precision of measurement should be such as to permit the detection of a change of 0.1 *μ*v in the difference between the two emf’s, for any value of this difference up to, say, 1,000 to 2,000 *μ*v, and the accuracy of measurement should be of the same order of magnitude.
- The value of the unknown emf should be indicated directly, regardless of whether it is higher or lower than the emf of the reference cell, without attention to this point by the operator, and the manner of indicating the result should be direct and unambiguous in order to minimize the liability of errors in writing down the observed value.
- The reference cell should be used only to oppose the unknown cell, and means independent of it should be used to check the magnitude of the battery current through the potentiometer.
- The final balancing of the two emf’s should be a continuous process, so that the deflection of the galvanometer can be reduced to zero. In other words, it should not be necessary to interpolate between two deflections of the galvanometer to obtain the final value. Interpolation is objectionable in standard-cell comparisons not only because it requires time and mental effort with liability of error, but also because it involves the passing of current in both directions through each of the cells. The passage of even small currents through a standard cell against its emf is to be particularly avoided.

### 4. METHOD WHICH MEETS THE REQUIREMENTS

**Figure 1:**
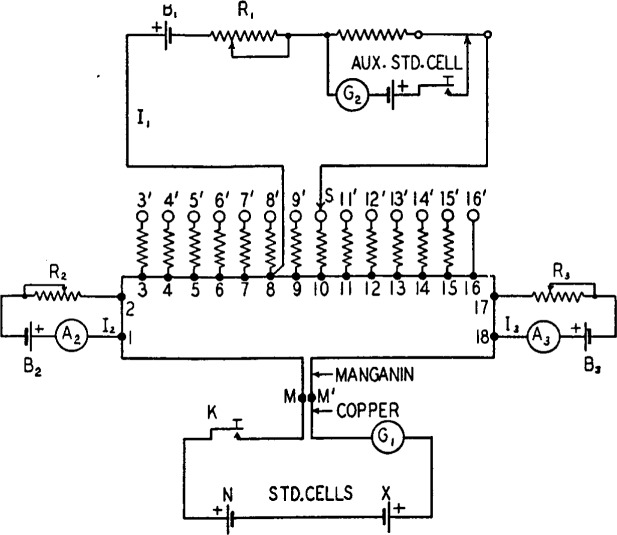
Diagrammatic plan of circuits illustrating the principle of the Potentiometric part of the standard-cell comparator.

The principal features of the method which has been developed to meet the preceding requirements may be explained by reference to figure 1. The reference cell *N* and the unknown cell *X* are opposed through a galvanometer *G*_1_ and a key *K* to the fall of potential in a continuous loop of manganin wire. Taps brought out from the points *3, 4, 5 __ __ __ 16*, divide the part of the wire between the points 3 and *16* into sections of equal resistance. The resistances of the sections *1–2* and *17–18* may be arbitrarily chosen and the small resistances of the sections *2–3* and *16–17* are immaterial. The junctions of the ends of the manganin wire with the copper wires leading to the galvanometer and the key are close together and are protected (by means not shown) from inequality of temperature. The manganin-copper circuit is thus thermoelectrically neutral to a high degree and contains no sliding contacts except those of the key *K.*

A current *I*_1_ from a battery *B*_1_ is regulated by the rheostat *R*_1_ to a definite value in the usual manner by reference to an auxiliary standard cell. This current enters the manganin wire at the tap point *8* and leaves it by the slider *S.* The substitution resistances between the tap points *3, 4,5 __ __ __ 15* and the corresponding contact studs, *3*′, *4*′, *5*′ *__ __ __ 15*′ maintain the resistance between *S* and the point *8* constant for all settings at the value it has with *S* on the stud *16*′. Let the current *I*_1_ have such a value that the drop of potential which it sets up in any one of the sections *3–4, 4–5, 5–6 __ __ __ 15–16* will be 100 *μ*v. Assume initially that the currents *I*_1_ and *I*_3_ through the sections *1–2* and *17–18* are zero. Then with the slider on the stud *8*′ no current flows in the manganin wire (with the key *K* open) and no difference of potential is set up in it. With the slider set successively on studs *9*′, *10*′, *11*′ *__ __ __ 16*′, the current flowing from the point *8* to the points *9, 10, 11 __ __ __ 16* will cause a drop of potential of 100, 200, 300 __ __ __ 800 *μ*v, of such polarity that if the emf of cell *X* is 100, 200, 300 __ __ __ 800 *μ*v lower than that of cell *N*, the sum of the drop of potential and the emf of cell *X* will be equal to the emf of cell *N.* If *S* be set on the studs *7*′, *6*′, *6*′ *__ __ __ 3*′, there will be a drop of potential of 100, 200, 300 __ __ __ 500 *μ*v in the opposite sense, which will produce the condition of balance if the emf of cell *X* is 100, 200, 300 __ __ __ 500 *μ*v higher than that of cell *N.*

If steps of 100 *μ*v were sufficiently small, or if interpolation between steps were admissible, the sections *1–2* and *17–18* of the manganin wire would be superfluous. To make it possible to balance cell *N* against cell *X* when their difference is not an exact multiple of 100 *μ*v requires the use of sections *1–2* and *17–18*, each with an associated ammeter, dry cell, and regulating rheostat. It would be possible to obtain the result by using only one of these two sections, but to carry out the direct-reading feature of the apparatus conveniently, both are used. Their functioning will now be explained.

If the emf of cell *X* is exactly 200 *μ*v less than that of cell *N*, for example, the slider *S* will be set on the stud *10*′ and the drop of potential in the two sections *8–9* and *9–10* will just produce the condition of balance. However, if the cell *X* has any intermediate value between 100 and 200 *μ*v lower than that of *N*, it is possible to adjust a current *I*_3_, flowing through the section *17–18*, so that the auxiliary drop of potential thereby introduced restores the condition of balance. An increase in the value of *X* requires an increase in *I*_3_, and it is convenient to mark the scale of the ammeter *A_3_* to indicate directly the drop of potential in the section *17–18.* A duplicate auxiliary circuit is provided which may be used to send a current *I*_2_ through the section *1–2.* This functions in the same manner as the auxiliary circuit for *I*_3_, except that the two currents flow in opposite directions through the manganin wire. For any value of the difference of emf of *N* and of *X*, the slider *S* may be set on one or the other of the two main-dial studs, between which the balance point lies, and an exact balance may be obtained (1) using auxiliary current *I*_3_ only; (2) using auxiliary current *I*_2_ only; or (3) using both *I*_2_ and *I*_3_. This last procedure is the one actually used, the values and directions of *I*_2_ and *I*_3_ being so chosen as to make the indicator *B* read directly the last two figures (that is, those in the fifth and sixth decimal places) of the value of *X* in terms of *N.* Briefly, this is accomplished by setting the current *I*_2_ at the value which produces a drop of potential in the section *1–2* equal to the last two figures in the value of *N.* The direction of the current *I*_2_ through the section *1–2* is such as to make the potential of the point *2* lower than that of *1*. The combination of the cell *N* and the section *1–2* is therefore equivalent to a reference cell having the same numerical value of emf as *N*, to and including the digit in the fourth decimal place, but having zeros in the fifth and sixth places. When the condition of exact balance is obtained by setting the slider *S* and adjusting the current *I*_3_, the reading of the ammeter *A*_3_ (in microvolts) will therefore be the fifth and sixth figures in the value of *X.* The manner in which the comparator gives directly the other figures for the value of *X* is given in the following section on the direct-reading feature of the comparator.

Although the direct-reading procedure just outlined involves the added cost of the ammeter *A*_2_ and its rheostats, it speeds up the work of comparing cells and tends to avoid errors which may arise when figures must be added or subtracted to obtain the final result.

Each of the sections *1–2* and *17–18* of the manganin wire *MM*′, with its associated milliammeter and adjustable current supply, is really a potentiometer operating according to Poggendorff ‘s^1^ little-used “second method.” The first application of this method as the basis for a commercial instrument for specific applications appears to have been made by Lindeck and Rothe^2^ at the Reichsanstalt in 1899. The instrument was developed^3^ with the cooperation of Siemens and Halske, who placed it on the market. The use of a “second-method” potentiometer as one component element of a potentiometer, supplementing one or more other “decades” of higher denominations, as is done for the first time in the standard-cell comparator, was suggested by the writer in a paper on potentiometers before the International Electrical Congress of 1932 at Paris. To avoid the usual but occasionally illogical expression “decade” the writer prefers to speak of certain structural components of a potentiometer as “elements”. For the lack of a suitable short descriptive term the expression “Lindeck-Rothe element” will be used in referring to the two “second-method” potentiometers which serve as “elements” of the standard-cell comparator.

## III. DIRECT-READING FEATURE OF THE COMPARATOR

**Figure 2:**
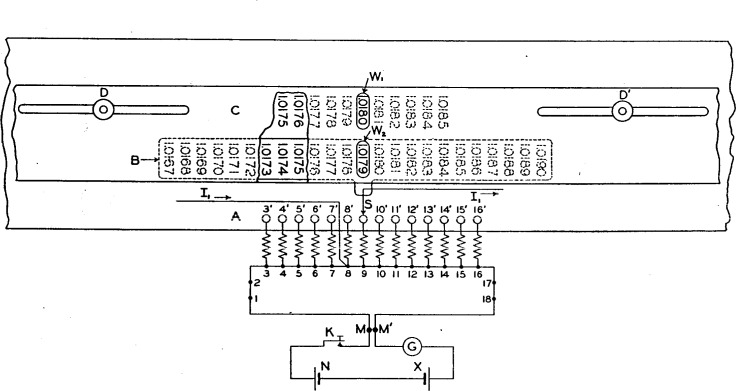
Diagram illustrating the principle of operation of the simple mechanical computing device which makes the standard-cell comparator indicate directly the emf of the unknown cell.

The potentiometer briefly outlined in the preceding section actually measures only the difference between the emf of the reference cell and that of the unknown cell. The mechanical computing arrangement which makes it possible to read directly the emf of the unknown cell is shown diagrammatically in figure 2, in which *A* may be thought of as a flat plate of insulating material carrying the contact studs *3*′ to *16*′ and marked with a scale of values of the reference emf ranging from 1.0175 to 1.0185 volts. The scale plate *B* is attached to the slider *S* and moves with it and is marked with a scale of values of unknown emf ranging from 1.0167 to 1.0190 volts. The opaque screen *C* covers both of the scales, with the exception of one value on the reference scale and one on the unknown scale, which appear, respectively, in the window openings *W*_1_ and *W*_2_. The screen may be adjusted laterally and clamped by the nuts *D D*′ so as to expose any desired one of the 11 values on the reference scale. For any position of the slider *S* and its associated scale plate *B* the value of unknown emf appearing in the window *W*_2_ will obviously depend upon the lateral adjustment of the screen. The correlation of the two scales is based upon the following facts: (*a*) When the slider *S* is on the stud *8*′ the current *I*_1_ does not flow through any part of the manganin wire *MM*′; (*b*) the flow of *I*_1_ through the manganin wire toward the right from tap point *8* sets up a difference of potential between *M* and *M*′ which aids the emf of cell *X* to balance that of cell *N;* (*c*) the flow of *I*_1_ through the wire *MM*′ toward the left from tap point *8* sets up a difference of potential between *M* and *M*′ which aids the emf of cell *N* to balance that of cell *X.* It follows from (*a*) that when the slider *S* is on the stud *8*′ the value of unknown emf appearing in *W*_2_ must be the same as the value of reference emf appearing in *W*_1_, for all positions of the screen *C.* Since the potential difference which *I*_1_ sets up in each of the sections *3–4, 4–5*, etc., of *MM*’ is 0.0001 volt, it follows from (6) that with the screen *C* clamped in any position the values of unknown emf which appear successively in *W*_2_ as *S* is set on studs *9*′, *10*′ *__ __ __ 16*′ must be 0.0001, 0.0002 __ __ __ 0.0008 volt lower than the value of reference emf which appears in *W*_1_. Similarly, with the screen *C* clamped in any position the values of unknown emf which appear successively in *W*_2_ as *S* is set on studs *7*′, *6*′ *__ __ __ 3*′ must be 0.0001, 0.0002 __ __ __ _ 0.0005 volt higher than the value of reference emf which appears in *W*_1_. In the actual use of the comparator, the screen is set to expose in *W*_1_ the certified or assumed emf of the reference cell, and consequently the value which appears in *W*_2_, when the condition of balance exists, must be the emf of the unknown cell.

A part of the laterally adjustable screen *C* is broken away to show figures on the reference scale (above) and on the scale of values of unknown emf. This latter scale is attached to the slider *S* and moves with it. *W*_1_ and *W*_1_ are the window openings through which one value on each scale may be seen.

In the preceding explanation it has been assumed for simplicity that 0.0001 volt is the limit of sensitivity of the apparatus; consequently no provision is shown in figure 2 for passing measured currents through the sections *1–2* and *17–18* of the manganin wire *MM*′, forpurposes previously explained.

The screen *C* as shown in figure 2 is long enough to cover all of the values marked on the scale plate *B* (except the one appearing at the window *W*_2_) for all positions of *B.* This is not a necessary feature, and *C* could be reduced to a size sufficient merely to accommodate the window openings *W*_1_ and *W*_2_. The arrangement shown, however, has the operating advantage that when balance is obtained only one value of unknown emf can be seen, namely, the one which is to be recorded.

In the comparator as actually constructed the contact studs are more numerous than in figure 2, and are arranged in the form of an incomplete circle. The reference scale is marked on the hard-rubber top, and the scale of values of unknown emf is marked on a hard-rubber disk rigidly connected with the rotatable contact brush. The screen is a metal cover or turret which encloses the studs, contact lever, and rotating scale and has, at opposite ends of a diameter, the two window openings which correspond to *W*_1_ and *W*_2_.

## IV. DESCRIPTION OF THE COMPARATOR

### 1. DIAGRAMMATIC PLAN OF CIRCUITS

**Figure 3:**
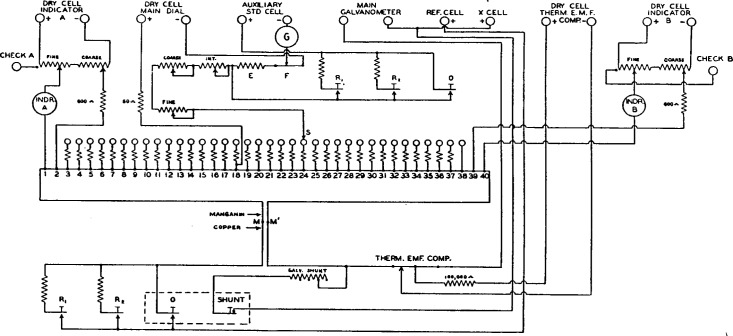
Complete diagrammatic plan of circuits of standard-cell comparator.

For the sake of simplicity a number of features of the comparator are not shown in figure 1. All the essential parts are indicated diagrammatically in figure 3, which is a development from figure 1. The manganin wire *MM*′ is divided into 35 dial sections of 0.05 ohm each by tap wires from the points *3, 4 __ __ __ 38*; sections *1–2* and *39–40* are also of 0.05 ohm each, and the small resistances of sections *2–3* and *38–39* are immaterial.^4^ A current of 2 milliamperes from the positive pole of a dry cell connected to the terminals marked “dry cell main dial” enters the tap wire attached to the point *18* on the manganin wire and flows to the right or the left through the manganin wire, or back to the cell without entering the manganin wire, according to the position of the slider *S*. The fall of potential in the manganin wire is 100 *μ*v per section. The instruments marked “Indr. A” and “Indr. B” for measuring the currents in the sections *1–2* and 3*9–40* of the manganin wire are actually milliammeters, but their 100-division scales are marked “microvolts” to indicate the value of the fall of potential in sections *1–2* and 3*9–40.* The algebraic sum of the three potential drops in the manganin wire is opposed, through keys and a galvanometer, to the resultant emf of the reference cell and the unknown cell connected in opposition.

### 2. MAIN-DIAL RESISTOR

The copper-manganin junctions at the ends of the manganin wire *MM*′ (fig. 3) must be carefully protected against inequality of temperature which would set up a thermal emf and cause an error in the measurement. For this reason the manganin wire was inclosed in a bakelite box supported from the under side of the hard-rubber top of the comparator. This construction shelters the resistor effectively from heat radiation and conduction. Furthermore, the two copper-manganin junctions are placed very close together. The thermal emf of a copper-manganin junction is about 1 to 2 *μ*v per ° C, consequently the two junctions must not differ in temperature by 0.05° C., if the parasitic emf in this part of the apparatus is to be kept below 0.1 *μ*v.

The manganin wire used was no. 16, A.W.G. (diameter, 0.051 inch = 1.3 mm). After being wound into a helix the wire was annealed at a red heat. The copper tap wires were attached to it by hard soldering.

### 3. MAIN DIAL

**Figure 4:**
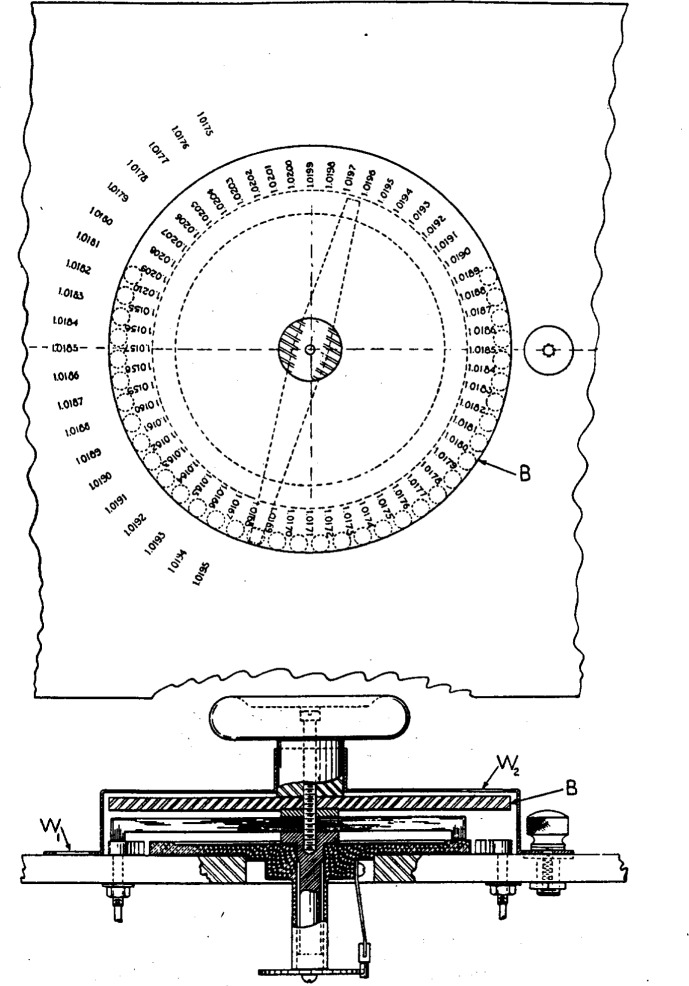
Above, central portion of the hard-rubber top of the comparator, carrying a scale of values of the reference emf ranging from 1.0176 to 1.0195 volts. The handle for operating the main-dial contact brush, and also the main-dial screen, have been removed to show the scale of values of unknown emf marked on the disk B, which moves with the contact brush. Below, view of the main dial, partly in cross section, showing the location of the two window openings *W*_1_ and *W*_2_ for the scales ot reference emf and unknown emf, respectively.

The main dial consists of 36 brass studs placed on a circle which if filled would contain 56 studs. (See fig. 4.) This unusual arrangement is necessary for the following reasons: The lowest value marked on the reference scale is 1.0175 volts; since there are 20 steps of 0.0001 volt each to the right of tap point *18* on the manganin wire *MM*′ (see fig. 3), the lowest value of unknown emf which can be measured, using a reference emf of 1.0175 volts, is 1.0175 − 0.0020 = 1.0155 volts. The highest value marked on the reference scale is 1.0195 volts, and with a reference emf of this value the 15 sections of the wire *MM*′ to the left of tap point *18* make it possible to measure a maximum unknown emf of 1.0195 + 0.0015 = 1.0210 volts. Thus the circular scale *B* of values of unknown emf which is to rotate with the contact brush in figure 4 must bear the 56 numbers, 1.0155, 1.0156 __ __ __ 1.0210. These figures, however, do not represent the usual working range of the comparator, which is discussed later under the heading of operating limits.

Even after the impending change in the unit of emf becomes effective, it is not expected that any occasion will arise for measuring an emf much greater than 1.0195 (new) volts. In other words, when a reference cell having an emf of 1.0195 (new) volts will be used, the slider *S* (fig. 3) will probably never be placed more than a few steps to the left of tap point *18.* A choice had to be made between two courses: First, to decide that no values of unknown emf greater than, say 1.0198 (new) volts, would ever be measured, and that consequently the part of the rotating unknown-emf scale of the main dial corresponding to values of 1.0199 to 1.0210 volts, inclusive, should be left blank; or second, that all of these probably unnecessary numbers should be marked on the scale, to indicate the position of the main-dial contact brush. The latter course was chosen.

**Figure 5:**
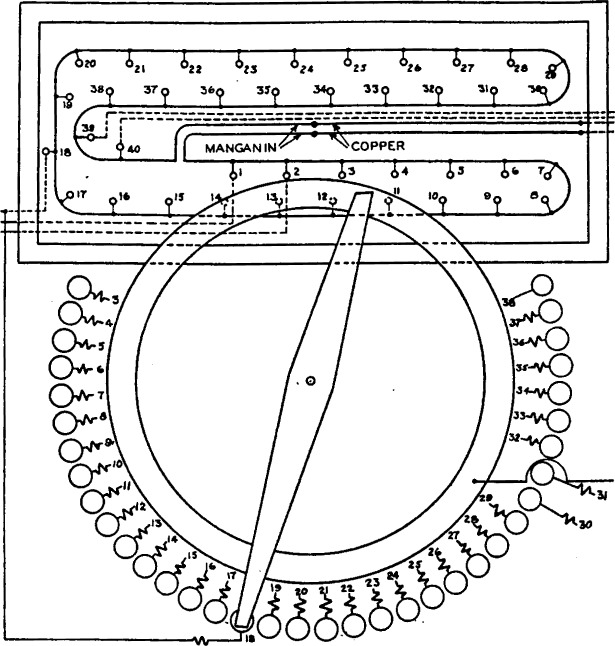
Semidiagrammatic plan view of main resistor and of the main-dial contact studs, brush, and ring. The substitution coils connected to the dial studs are indicated; their free ends are actually joined to the correspondingly numbered tap points on the main resistor by connecting wires which are omitted for clarity.

Figure 5 shows senudiagrammatically the essential parts of the main dial and of the manganin wire in its bakelite enclosure. Each of the contact studs (except no. 38) carries a substitution coil which maintains constant the total resistance in the circuit through which current is supplied to the main-dial resistor. In figure 5, to avoid confusion, the wires from the tap points on the main-dial resistor to the substitution coils are not shown but are indicated by numbers; thus from the tap points *3,4 __ __ __ 37*, wires run to the free ends of substitution coils *8, 4 __ __ __ 37.* Tap point *88* is connected directly to stud *38.*

### 4. LINDECK-ROTHE ELEMENTS

The current through each of the main-dial resistor sections *1–2* and *39–40* (see fig. 3) is measured by a milliammeter designed especially for the purpose. The full-scale current is 2 milliamperes. The scale has 100 divisions and the position of the knife-edge pointer may be accurately read with the aid of a parallax mirror. The current for each element is supplied by a dry cell and is adjusted by a coarse rheostat of 15 steps in series with a fine rheostat for close adjustment. The contact slider of each fine rheostat is ordinarily constrained by a stop to run only over the winding. Near the end of the winding which runs to the positive pole of the dry cell is a contact stud leading to a binding post. These binding posts are marked “check A” and “check B”, respectively. They are for use when it is desired to check the accuracy of the milliammeters, which is done as follows: After releasing the stop on the slider of the fine rheostat at the extreme left of figure 3, the slider is set on the stud leading to the binding post marked “check A.” Then the wire from the positive pole of the main-dial dry cell which normally sends a current to tap point *18* on the main-dial resistor is detached from its usual binding post and clamped in the one marked “check A.” With this special arrangement the main-dial current flows through indicator *A* into the main-dial resistor at tap point *1*, out at the slider *S*, back to the dry cell through the three regulating rheostats marked “fine”, “int.” and “coarse”, and the coil *E* and slide wire *F*, which serve, in connection with an auxiliary standard cell, to adjust the main-dial current to exactly 2 milliamperes. This latter adjustment having been made, the pointer of the milliammeter should be deflected exactly 100 divisions. If the deflection differs somewhat from this value, it is to be brought to 100 divisions by adjusting a magnetic shunt which varies the magnetic flux in the air gap of the milliammeter. This may be done without removing the case of the milliammeter.^5^ The same procedure may be applied to check indicator *B* at the right (fig. 3), but in this case the + wire from the main-dial dry cell is to be connected to the special binding post marked “check B.”

The 50-ohm coil in the wire joining tap point *18* of the main-dial resistor to the + binding post of the pair marked “dry-cell main dial” has approximately the same resistance as either of the two indicators. Consequently the shifting of the wire from the positive pole of the dry cell for the main dial from its usual binding post to either the check A or check B binding post does not appreciably alter the total resistance in the path of the main-dial current. By the above method of adjusting the indicators to read correctly, their readings, as well as those derived from the position of the slider *S*, are in terms of the unit of emf embodied in the auxiliary standard cell. Therefore the entire result of a measurement, with the comparator, of the difference in emf of an unknown cell and a reference cell is referred to the unit of emf in terms of which the auxiliary standard cell is certified.

An important feature of the Lindeck-Rothe element as a component of a potentiometer is the fact that its indications may readily be made independent of changes of room temperature.^6^ It shares with the slide wire the advantage of a continuous change of potential difference which makes interpolation unnecessary. Unlike the slide wire, however, it may readily be made as thermofree as desired.

### 5. THERMOFREE^7^ GALVANOMETER KEYS

The three keys marked *“R*_1_*”*, *“R*_2_*”*, *“O”* in the upper part of figure 3 and those at the bottom marked *“R*_1_*”, “R*_2_*”* are of ordinary construction. The conditions under which they operate are such that any thermal emf at the key contacts is negligible. The keys at the bottom marked *“O”* and “shunt” must be highly thermofree, namely, they must show no thermal emf as great as 0.1 *µ*v, even under relatively adverse thermal conditions.

Ordinary keys contain combinations of metals (for example, brass or bronze with platinum) which may develop a thermal emf of one or more microvolts under usual working conditions.^8^ Their construction is usually such that heat may be transferred from the observer’s hand to the contact points. Three expedients to minimize thermal emf have been used in the design and construction of the keys *O* and shunt, namely (1) the choice of materials for the key springs and the contacts which are thermoelectrically near to copper; (2) the design of the key to be “thermoelectrically astatic”, that is, with soldered junctions and abutting contact points symmetrical with respect to such heat flow as cannot be avoided; and (3) the inclosure of the key in a shell made of material of good thermal conductivity which distributes any heat which reaches it, this heat being then transferred uniformly from the inside of the shell to the key.

**Figure 6:**
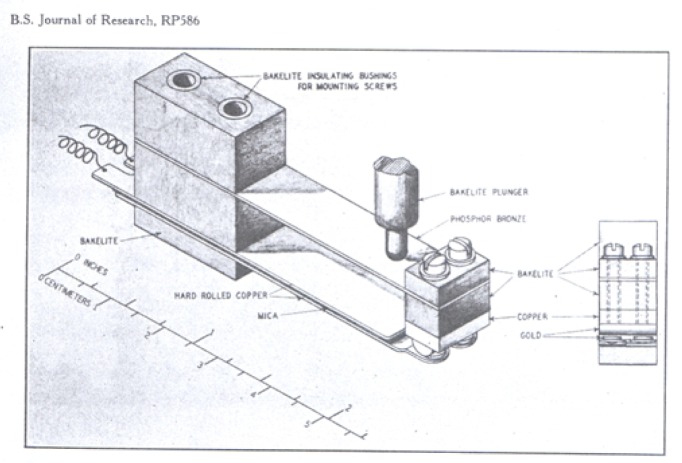
Galvanometer key embodying refinements to minimize thermal emf.

The thermofree keys used in the standard-cell comparator are of a type developed for a potentiometer used with thermocouples in measuring very small temperature differences and requiring the reduction of “parasitic” thermal emf to an even lower value (0.02*µ*v) than in the comparator. They have springs of hard-rolled copper and contact points of United States coin gold.^9^ The “thermally astatic” arrangement of the operating parts of the key marked *“O”* is shown in figure 6. This key,^10^ and the very similar one marked “shunt”, which is normally closed, are inclosed in a box of cast aluminum 0.1 inch (2.5 mm) thick.

### 6. AUXILIARY GALVANOMETER

In most potentiometers a single galvanometer is used for two purposes; first, in series with a standard cell for adjusting the auxiliary (battery) current through the potentiometer to its normal value, then in series with the emf under measurement. In most cases this dual use of the galvanometer is convenient and satisfactory, but for the standard-cell comparator it was considered inadvisable, and an inexpensive but adequate auxiliary galvanometer was built into the potentiometer, permanently in series with the auxiliary standard cell. The cost of this auxiliary galvanometer is at least as low as that of a highly thermofree changeover switch which would be necessary to enable the main galvanometer to do double duty; its period is only one third of the period of the main galvanometer; it saves the main galvanometer from occasional (accidental) large deflections; the auxiliary galvanometer is sufficiently sensitive, the main galvanometer would be needlessly over-sensitive, leading to waste of time in the effort to adjust the auxiliary current to a needless degree of precision.

### 7. SHUNT COIL FOR MAIN GALVANOMETER

This coil, indicated in figure 3, is connected, through the key marked “shunt”, across the terminals of the main galvanometer. This key is normally closed. The shunt coil is of copper wire to reduce to a negligible amount the possibility of thermal emf, and is wound in a single layer on micanite cards and varnished to exclude moisture. It has a total resistance of 1,400 ohms, and taps are brought out at 800, 1,000, and 1,200 ohms. The main galvanometer to be used eventually with the comparator is to be critically damped when the external resistance is 1,200 ohms, which was assumed as an average value of the resistance of two saturated cadmium cells in series. The other values of resistance of the shunt coil were provided to take care of possible deviation of the galvanometer from this intended value of external resistance for critical damping. One of the purposes of the shunt coil is to prevent oscillation of the galvanometer coil when the keys *R*_1_, *R*_2_, and *O* are not depressed. The coil has also another important use which is explained in the next paragraph.

### 8. THERMAL EMF COMPENSATOR

This is a simple slide rheostat wound with copper wire and connected in series with the main galvanometer (see fig. 3). A current of about 15 *µ*a from a dry cell enters this winding at its central point and leaves by the slider. If the slider is set on the central point of the copper winding, the current flows back to the dry cell without having passed through any part of the copper winding and therefore without producing any drop of potential in that winding. By setting the slider away from the central point, a small, adjustable drop of potential may be introduced into the galvanometer circuit to neutralize the effect of any parasitic emf in the galvanometer and the wires connecting it to the comparator. By depressing the key marked “shunt” the user can determine at any time whether any appreciable parasitic emf is present. Any such emf will have maintained a corresponding deflection of the galvanometer, and when the shunt key is depressed the galvanometer coil will assume its open-circuit zero position. To neutralize any such undesired emf the observer simply manipulates the slider of the thermal emf compensator until no motion of the galvanometer coil ensues when the shunt key is depressed. This operation may be performed regardless of the position of the main-dial contact brush or of the currents through the indicators, and regardless of whether standard cells are, or are not, connected to the comparator for test.

By replacing the 100,000-ohm resistor of the thermal emf compensator with one of higher or lower resistance, the number of microvolts per angular degree of rotation of the slider can be varied to suit the needs of the particular galvanometer and its environment.

The thermal emf compensator, used as just described, takes no account of parasitic emf within the comparator. The design of the comparator and the materials used in its construction are such that the internal parasitic emf under any reasonable operating conditions will be much less than 0.1 *µ*v. This point may properly be checked in testing a new instrument for acceptance, the procedure being as follows: The reference cell and the cell under test are to be disconnected from the comparator and the posts marked “+Ref” and “+X Cell” are to be joined by a copper wire; then one wire is to be detached from each of the three dry cells which supply indicators *A* and *B* and the main dial (see fig. 3). The disconnection of these three dry cells definitely insures that no current flows through the manganin wire *MM*′ of the main dial. The shunt key is then to be depressed and the slider of the thermal emf compensator manipulated until the galvanometer coil no longer moves when this key is depressed. External parasitic emf having thus been compensated, the depression of the shunt key and the adjoining key marked *“O”* will show whether any parasitic emf exists in the interior of the comparator.^11^ In making such a test, it should be remembered that the parasitic emf in the galvanometer may vary irregularly, and the check for its presence and its compensation should be repeated often enough to make sure that an apparent parasitic emf in the comparator is not actually a change in the outside one. It is also important to realize that a parasitic emf may result from other than thermoelectric causes, such as leakage which may take place from nearby direct-current power and battery circuits. High insulation of all parts of the cell-comparison apparatus and its wiring, and guarding (if necessary) by methods analogous to the classical Price procedure^12^ are the remedies.

The two binding posts for the main galvanometer and the ones marked “+Ref. Cell” and “+X Cell” have contact surfaces of copper to avoid thermal emf. This precaution is unnecessary for the other binding posts. Thermal emf at the various sliding contacts in the comparator cannot affect the accuracy of measurement.

### 9. DRY CELLS AS SOURCES OF CURRENT

The use of storage cells to supply current to the comparator has not been contemplated, and the regulating rheostats have such ranges of resistance as to permit the use of dry cells having an initial emf of not over 1.62 volts and a cut-off point (at which the cells are to be discarded) of 1.30 volts. The reasons for providing for dry cells only are that the dry cell is the cheapest and cleanest source of current for the purpose, is readily obtainable, and is adequate for the purpose.

Although single-pole switches might have been included in the instrument to open the circuits of three of the dry cells when the comparator is not in use, this was considered an unnecessary refinement. The current supplied by each of the two indicator cells is from 2.7 to 4.5 ma, depending on the deflection, and the main-dial dry cell supplies 2 ma. Left in these circuits continuously, no. 6 dry cells^13^ of good quality may be expected to last about 10 months, the actual time depending considerably on the average room temperature. Even if the cells were switched off when not in use, the ordinary deterioration on open circuit would prevent any great extension of their useful life. If the user wishes to open the circuit of these three cells during long periods of disuse, this may be readily done by detaching one wire at each cell. There is no reason whatever for doing this with the cell which supplies the minute current for the thermal emf compensator.

### 10. ARRANGEMENT OF PARTS OF THE COMPARATOR

**Figure 7:**
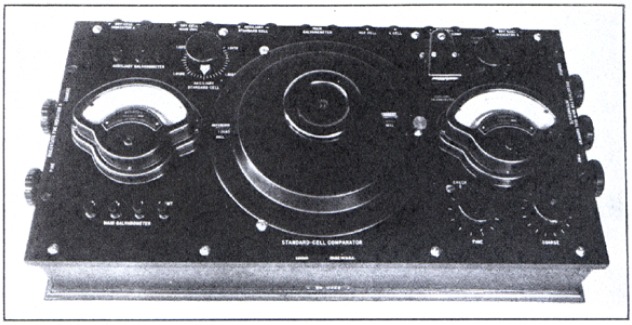
The standard-cell comparator from a photograph.

Figure 7 shows a view of the standard-cell comparator.^14^ The arrangement of the major components of such an apparatus should be conducive to convenience in manipulation and in reading the result of a measurement. Both of these qualities tend to expedite the work and to minimize the probability of errors. Some of the ideas leading to the particular arrangement adopted may be briefly stated.

The main dial is centrally located and is intended to be operated with the right hand while the left manipulates the main-galvanometer keys. The reference-cell indicator, *A*, infrequently observed, and its two rheostats, infrequently manipulated, are placed at the extreme left. The reference-cell window in the screen^15^ of the main dial is placed as near the reference-cell indicator as possible, to assist in conveying the idea that the sum of their readings is to be set to equal the known or assumed value of the emf of the reference cell. The much-used indicator *B* and its rheostats are placed at the right, and the reading in the unknown-cell window, nearby, plus the reading of indicator *B* equals the value of the unknown emf. The three rheostats for the main-dial current, occasionally used, are placed at the right-hand end of the comparator. The auxiliary galvanometer, thermal emf compensator, auxiliary standard-cell dial and the three keys in the circuit with the auxiliary galvanometer, all used only occasionally, are placed behind the indicators and the main dial.

The auxiliary galvanometer has a vertical scale readily seen by the operator without any change of position. This galvanometer has an interchangeable suspended-coil system which may be readily removed for repairs or replacement.

**Figure 8:**
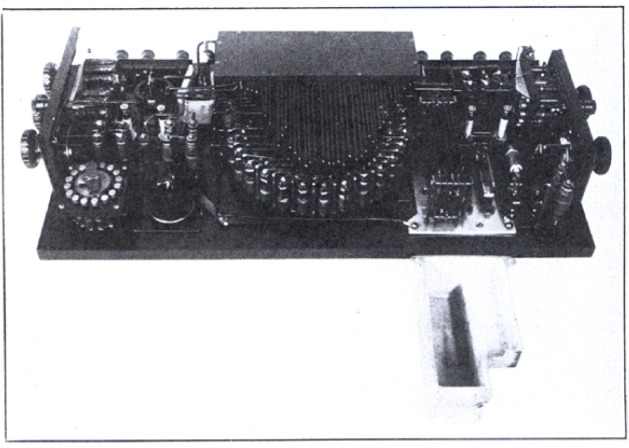
Interior of the standard-cell comparator. Centrally located at the top is the bakelite box containing the main-dial resistor. The tap wires from this resistor extend downward to the substitution coils of the main dial. The aluminum box which normally encloses the thermofree keys and the copper shunt coil been removed to show these details.

Figure 8 shows a view of the interior of the comparator. Centrally located at the top is the bakelite box containing the main-dial resistor. The tap wires from this resistor extend downward to the substitution coils of the main dial. The various regulating rheostats may be clearly seen. The aluminum box which ordinarily encloses the two thermofree keys and the copper shunt coil for the main galvanometer has been removed to show these details.

The over-all dimensions of the comparator with cover are 13 inches (330 mm) by 26 inches (660 mm) by 8.1 inches (206 mm), and its weight is 42 pounds (19 kg).

## V. OPERATION OF THE COMPARATOR

### 1. OPERATING PROCEDURE

The procedure of setting up and operating the comparator may be outlined as follows: A dry cell is connected to each of the four pairs of binding posts so marked. A cadmium standard cell, which may conveniently be of the unsaturated type, is connected to the binding posts marked “auxiliary standard cell”, and the auxiliary standard cell dial is set to the known value of this cell. The negative pole of the standard cell under measurement is joined to the negative pole of the reference cell, and their positive poles are connected to the correspondingly marked binding posts of the comparator. The external galvanometer is connected to the comparator binding posts marked “main galvanometer”. For the most precise measurements of which the comparator is capable this galvanometer should be a high-grade instrument of constants appropriate for the purpose.^16^

If the comparator is being set in operation for the first time, it is desirable to check the accuracy of the two indicators^17^ as described in the section above on Lindeck-Rothe elements, and to carry out the test for internal parasitic e.m.f. as described in the section on thermal emf compensator.

The screw which clamps the main-dial screen in position is then released, the screen rotated until the number appearing centrally in its reference-cell window is the known (or assumed) value of the reference cell, to and including the figure in the fourth decimal place, and the screen is firmly clamped in this position. By means of its two rheostats, indicator *A* is then made to read the number of divisions (microvolts) corresponding to the number formed by the fifth and sixth digits of the decimal part of the reference-cell value.^18^ After this, the main-dial current is adjusted by means of the three regulating rheostats at the right-hand end of the comparator until the auxiliary galvanometer shows no deflection with its zero-resistance key depressed.

The test for the presence of intruding emf in the external part of the measurement circuit is then made, and any such emf compensated, as described above under the heading “Thermal emf compensator.” All is then in readiness for the actual comparison of the cells. Depression of the main-galvanometer key R_1_ closes the measurement circuit through a protective resistance^19^ so high that for normal connection of the unknown cell and the reference cell the deflection will be small; seldom greater than 10 mm. The main-dial handle is then to be turned until the deflection is reduced to a fraction of 1 mm; then the main-galvanometer key *R*_2_ is to be used instead of *R*_1_, and two adjacent settings of the main dial are to be found, between which the direction of the galvanometer deflection changes. The corresponding numbers which appear in the screen window marked “unknown cell” will differ by 0.0001 volt. Leaving the dial with the smaller of these two numbers appearing in the window, the operator manipulates the coarse rheostat and the fine rheostat near indicator *B*, meanwhile testing for the condition of balance in the measurement circuit by tapping the main-galvanometer key *R*_2_, until the deflections become only a fraction of 1 mm; then the main-galvanometer keys *O* and Shunt are to the used, depressing them simultaneously, in attaining an exact balance. The value of the emf of the unknown cell, in terms of the value assumed for the reference cell, is then written down as the number appearing in the “unknown cell” window supplemented by the reading of indicator *B* in microvolts. A figure for tenths of a microvolt may be had by estimating tenths of a division in the deflection of indicator *B.*

### 2. OPERATING LIMITS

The comparator is designed to use a reference cell having any value of emf between the limits 1.017500 and 1.019100 international volts. When the impending change in the electrical units takes place, the emf of a cell which is 1.019100 international volts will be approximately 1.019500 absolute volts. Therefore, to avoid the necessity of altering the scales of the comparator, values are marked on the reference-cell scale up to 1.019500. With no restriction on the value of the reference emf within the limits 1.017500 and 1.019100 international volts, the comparator will measure any unknown emf and indicate its value directly over the range 1.017100 to 1.019100 international volts. With a reference emf of intermediate value the range of measurement is increased. For example, using a reference emf of 1.018300 volts, any unknown emf within the range 1.016300 to 1.019900 volts can be measured.

### 3. MISCELLANEOUS MEASUREMENTS OF EMF

The comparator has been designed for the optimal performance of its specific function, and is not nearly as convenient for ordinary emf measurements as a potentiometer of more conventional design. When circumstances require, however, it may be used for measuring any direct emf between zero and 1,600 *µ*v, and consequently may be used with a thermocouple for temperature measurements over a limited range. In this case the + and − terminals of the couple may be connected to the binding posts marked “+X cell” and “+Ref. cell”, respectively; the screen is set to expose any convenient number in the reference-cell window, 1.0190 volts for example, and indicator *A* is left at zero deflection. The unknown emf is balanced in the usual way by turning the main-dial handle and by deflecting indicator *B* until the main galvanometer shows no deflection. The numerical value of the unknown emf is found by subtracting the reference-scale reading from the entire “unknown” reading; that is, from the sum of the reading in the unknown-cell window and the reading of indicator *B.* For example, if the reference-scale reading is 1.0190, the unknown-scale reading 1.0202, and that of indicator *B* is 50 *µ*v, the unknown emf is 1.020250–1.0190 = 0.001250 volt. If the unknown emf under measurement in this way decreases through zero to a negative value,^20^ the measurements can be continued without any change of connections or of technique, down to −2,000 *µv.* For example, if the reference-scale reading is 1.0190, the unknown-scale reading is 1.0177, and that of indicator *B* is 20 *µv*, the unknown emf is 1.017720–1.0190= −0.001280 volt.

## VI. USE OF THE COMPARATOR AFTER THE VOLT IS CHANGED

In designing the standard-cell comparator for preassigned limits of reference emf and of unknown emf in terms of the “international volt”, the impending change to the “absolute volt” (equals 10^8^ cgs electromagnetic units of emf) was kept in mind, with the result that no structural alterations in the comparator will be required when the change becomes effective. Any cell suitable for use as a reference cell before the change in the unit may still be used after the change, and a similar statement applies to the unknown cells. The simplicity of the procedure for adapting the comparator to function on the new basis may be seen from the following statement. Assume that the effect of the change is to give the Weston cell a numeric greater by 0.000410 than the value assigned to it when the international volt was in force. Then on the date when the change in the units becomes effective, the auxiliary standard cell having thereby acquired a larger numeric, the setting of its dial is to be increased by 410 *µ*v. The value of the main-dial current for the condition of zero deflection of the auxiliary galvanometer will then need to be reduced about 1 part in 2,500, and consequently the two indicators, *A* and *B*, will theoretically need a slight readjustment of their magnetic shunts. Actually, the change required is only 0.04 division in a deflection of 100 divisions. This is about the possible limit of reading the coincidence of the pointer with a division line of the scale, and the readjustment of the indicators is therefore superfluous. The change in the setting of the auxiliary standard cell dial, the consequent readjustment of the main-dial current, and the change in the setting of the screen and of the reading of indicator *A* to accord with the revised value of the reference cell are the only things necessary in order to make the comparator give values of emf of cells under measurement in terms of the world’s new unit.^21^

The fact that the ohm will be changed when the volt is changed will not affect the use of the comparator because its measurements involve the ratio of definite parts of the manganin resistor *MM′* (fig. 3) to the resistance around which the auxiliary standard cell is balanced. If these parts of the main-dial circuit have the correct ratio, the unit of resistance in which they are adjusted is immaterial.

Washington, May 12, 1933.

## Footnotes

1 J. C. Poggendorff, Ann. der Physik und Chemie, vol. 54, p. 161, 1841.

2 Lindeck and Rothe, Zeitschrift für Instrumentenkunde. vol. 20, pp. 293–299, 1900; foreshadowed in the annual report of the Reichsanstalt, ibid., vol. 19, p. 249, 1899. Hoffman and Rothe, ibid., vol. 25, pp. 273, 278, 1905, described a recording potentiometer developed from the Lindeck-Rothe potentiometer, using the deflection-potentiometer principle.

3 Keinath, Elektrische Temperaturmessgeräte, 1923 edition, pp. 30–31.

4 Points *2* and *S* might theoretically be merged, and so could *38* and *39*; practically it would not be possible to connect two tap wires to points on the manganin wire which are at exactly the same potential.

5 To avoid the liability of mistake, or of possible meddling by unauthorized persons, it was made necessary to ose a small screwdriver to adjust the magnetic shunt.

6 The accuracy of direct-current milliammeters in which the entire current to be measured flows through the moving coil is almost independent of changes of the instrument temperature, because (1) no change in the distribution of the measured current between the moving coil and a shunt circuit around it can occur as a result of change of temperature; (2) the small temperature coefficient of magnetic flux density in the air gap (about 0.01 to 0.03 percent per °C.) tends to offset the temperature coefficient of rigidity of the springs, which is about 0.04 percent per °C. In the event that a shunt around the moving coil becomes necessary, however, it is still possible to arrange matters to make the indications of the instrument independent of temperature changes.

7 The word “thermofree” is offered as an arbitrary short equivalent of complete but unwieldy expressions such as “free from thermoelectric forces”, “thermal emf free”, “thermoelectrically neutral”, etc. Although it was suggested by the well-known German adjective “thermokraftfrei,” it was felt unnecessary to include the English equivalent of the syllable “kraft.”

8 Several makers of potentiometers have been using keys intended primarily for telephone purposes. Some telephone keys contain springs of very dissimilar alloys (evidently phosphor bronze and nickel-silver) and in consequence are extremely objectionable for use in potentiometers. There should be no difficulty in having telephone keys made up with bronze springs only, when the keys are to be used in potentiometer circuits.

9 The composition of United States coin gold is 90 parts gold to 10 parts copper. Its thermal emf against copper at room temperature is about 2*µ*v per °C.

10 For a detailed discussion of this key mechanism see paper by H. B. Brooks and A. W. Spinks on “A Multirange Potentiometer and its Application to the Measurement of Small Temperature Differences”, B.S. Jour. Research, vol. 9 (RP506), p. 781, December 1932.

11 In this part of the test the motion of the galvanometer coil will necessarily be heavily over-damped, and consequently the observer must give it plenty of time to complete its deflection. Because of the abnormally low external resistance the working sensitivity will be much greater than the normal value

12 W. A. Price, Elec. Rev. (London), vol. 37, p. 702, 1895; Ayrton and Mather, Phil. Mag., vol. 49, p. 343, 1900; W. P. White, Phys. Rev., vol. 25, p. 334, 1907; H. L. Curtis, Trans. Am. Inst. Elec. Engs., vol. 48, p. 1263, 1929.

13 “Number 6 dry cell” is an American trade designation (or a cylindrical dry cell which is 2.5 inches (63 mm) in diameter and 6 inches (152 mm) high, exclusive of the external cardboard jacket.

14 The comparator was built by the Leeds *&* Northrup Co. on the basis of the writer’s plans and specifications. The engineers of this company made many valuable suggestions for improvements in details of construction. The 2 special milliammeters for the Lindeck-Rothe elements were carefully developed to meet the writer’s requirements by the Weston Electrical Instrument Corporation.

15 The screen is shown in figure 7 in its intermediate angular position, corresponding to a reference-cell emf of 1.018S volts.

16 The galvanometer which was considered most suitable for precise comparisons of standard cells, and which has been kept in mind during the design of the comparator, has the following constants: Complete period, 9 seconds; external resistance for critical damping, 1,200 ohms; sensitivity with 1,200 ohms in series, 4 mm/*µ*v with a scale distance of 1 m.

17 The use of the word “indicator” or equivalent is necessary because although these two instruments are inherently milliammeters, they are used with their associated apparatus in emf measurements, and their scales are marked in microvolts.

18 By estimating tenths of a division, one could set the comparator for the reference-cell vatue out to the seventh decimal place.

19 In the comparator used at the Bureau of Standards this resistance is one half megohm. This high value is provided to prevent an excessive current from flowing through the two cells and the galvanometer in the event that the cell under test and the reference cell are inadvertently connected in series with the emf’s aiding. This would cause a resultant emf of over 2 volts to act where normally only a very small fraction of a volt is present.

20 This might happen, for example, if the difference in temperature of two baths, under measurement with a thermocouple, should change sign.

21 If it had not been necessary to provide for the change in the volt, the electrical features of the comparator would still have been as they now are, but the 36 studs of the main dial could have been placed on a smaller circle which if filled would contain 52 studs instead of 56.

